# Maternal Metformin Treatment Improves Developmental and Metabolic Traits of IUGR Fetuses

**DOI:** 10.3390/biom9050166

**Published:** 2019-04-29

**Authors:** Consolación Garcia-Contreras, Marta Vazquez-Gomez, José Luis Pesantez-Pacheco, Laura Torres-Rovira, Ana Heras-Molina, Teresa Encinas, Susana Astiz, Antonio Gonzalez-Bulnes

**Affiliations:** 1 SGIT-INIA, 28040 Madrid, Spain; garcia.consolacion@inia.es (C.G.-C.); jose.pesantez@ucuenca.edu.ec (J.L.P.-P.); torrerovi@gmail.com (L.T.-R.); delasheras.ana@inia.es (A.H.-M.); astiz.susana@inia.es (S.A.); 2 Faculty of Veterinary Medicine, Universidad Complutense de Madrid (UCM), Ciudad Universitaria s/n, 28040 Madrid, Spain; mvgomez@ucm.es (M.V.-G.); tencinas@vet.ucm.es (T.E.); 3 School of Veterinary Medicine and Zootechnics, Faculty of Agricultural Sciences, University of Cuenca, 010220 Cuenca, Ecuador

**Keywords:** intrauterine-growth-restriction, metformin, pregnancy, swine-model

## Abstract

Metformin is an anti-hyperglycemic drug widely used for the treatment of insulin resistance and glucose intolerance and is currently considered for preventing large-for-gestational-age (LGA) offspring in pregnant women affected by obesity or diabetes. Our hypothesis was the opposite—metformin may be used for improving the development of offspring affected by intrauterine growth restriction (IUGR) and preventing the appearance of small-for-gestational-age (SGA) neonates in non-obese and non-diabetic but malnourished pregnancies. The current study, performed in a swine preclinical model of IUGR by undernutrition, showed that fetuses in the treated group showed no significant increases in body-weight, but showed a significantly higher weight of the brain, the total thoracic and abdominal viscera, the liver, the kidneys, the spleen, and the adrenal glands. Maternal metformin treatment was also related to significant increases in the fetal plasma concentration of parameters indicative of glycemic (glucose and fructosamine) and lipid profiles (triglycerides). Overall, these results suggest a protective effect of the treatment on the developmental competence of the fetuses. These findings may be of high value for human medicine in case of maternal malnutrition, since metformin is a cheap drug easily available, but also in case of placental deficiency, since metformin seems to improve placental development and function.

## 1. Introduction

Metformin (3-(diaminomethylidene)-1,1-dimethylguanidine) is an anti-hyperglycemic drug widely used for the treatment of insulin resistance and glucose intolerance associated with obesity and diabetes [1]. The drug is currently considered as a potential agent for preventing large-for-gestational-age (LGA) offspring in pregnant women affected by obesity or diabetes [2,3]. These fetuses are exposed to high intrauterine concentrations of glucose through placental transport, which causes their prenatal development to accelerate. Hence, the hypothesis for using metformin is that the drug would prevent excessive birth-weight by improving maternal/fetal insulin sensitivity.

The hypothesis in which our study was based is just the opposite—metformin may be used for improving the development and performance of small-for-gestational-age (SGA) offspring affected by intrauterine growth restriction (IUGR) in non-obese and non-diabetic pregnancies but exposed to undernutrition. Such an idea may be strange at first glance, since metformin is a hypoglycemic agent, and fetuses affected by maternal undernutrition and IUGR have low glucose availability; hence, the use of metformin would be highly deleterious for their development and homeostasis. Moreover, metformin crosses the placenta and therefore acts not only indirectly on the maternal availability and transfer of glucose but also directly on the fetuses [4].

First, fetal glucose availability might be compromised indirectly by a strong decrease in maternal glucose availability. However, this is an unlikely event, because metformin would not cause overt hypoglycemia in the mother [5]. In fact, metformin lowers plasma glucose by improving insulin sensitivity (i.e., favoring uptake of glucose by the muscle and other peripheral tissues [6]). Hence, metformin may favor the use of glucose by IUGR fetuses and thus would improve their development. Our hypothesis would be supported by previous evidence addressing that metformin treatment in non-obese and non-diabetic individuals during juvenile stages induces an improvement in body development by favoring muscle deposition without fattening [7]. Similar effects might be exerted in prenatal stages.

With regards to direct effects on fetal glucose production, metformin lowers plasma glucose by decreasing its production at the liver, mainly as a result of a reduced gluconeogenesis [6,8]. However, the fetal liver has a glycolytic role during most of the pregnancy length and only assumes a gluconeogenic role shortly before birth [9], thus direct effects of metformin on fetal glucose production should be absent. 

In view of these considerations, our main objective was to determine the possible usefulness of a maternal metformin therapy to prevent and alleviate IUGR processes in the offspring. Such an objective was achieved by determining the effects of maternal metformin treatment on fetal development and metabolism of the offspring in a translational swine model of IUGR. The model was based on a maternal nutrient restriction of around 50% of the individual daily requirements during the last two thirds of pregnancy and induced a higher incidence of IUGR [10,11].

## 2. Results

### 2.1. Effects of Metformin Treatment on Maternal Weight and Metabolism

The assessment of maternal features in the control group (group C) and the metformin-treated group (group METF) at 100 days of pregnancy (Table 1) showed no significant effects of metformin treatment on body weight or on plasma parameters of metabolism of glucose (glucose, fructosamine) and lipids (triglycerides and total-, high-density lipoproteins cholesterol (HDL-c), and low-density lipoproteins cholesterol (LDL-c).

### 2.2. Effects of Metformin Treatment on Fetal Size and Metabolism

A total of 24 and 23 fetuses were obtained in the groups C and METF, respectively. Hence, the mean litter size was similar in both groups (8.1 ± 0.7 for C versus 8.4 ± 0.8 for METF). The sex ratio of the piglets was also similar and close to 1:1 in both groups, since 11 fetuses were females and 13 were males in the group C (45.8 versus 54.2%), while 13 fetuses were females and 10 were males in the group METF (56.5 versus 43.5%). There were no evident significant effects from litter size or sex on fetal size and metabolism.

The comparison of fetuses in the control and the treated groups showed no significant differences in weights, lengths, or widths of body and head (Table 2) in spite of numerically higher values for total-body-weight and carcass-weight in the group METF compared to those in group C. On the other hand, fetuses in group METF showed a significantly higher weight of the brain (*p* < 0.0005) and the total thoracic and abdominal viscera (*p* < 0.05). Moreover, significantly higher weights of liver, kidneys and adrenal glands (*p* < 0.05 for both), and spleen (*p* < 0.0005), as well as a trend for a higher weight of lungs (*p* = 0.07), were found in group METF compared to those in group C. The assessment of total placental weight showed a trend for higher values in the treated group (*p* = 0.06). 

**Figure 1:**
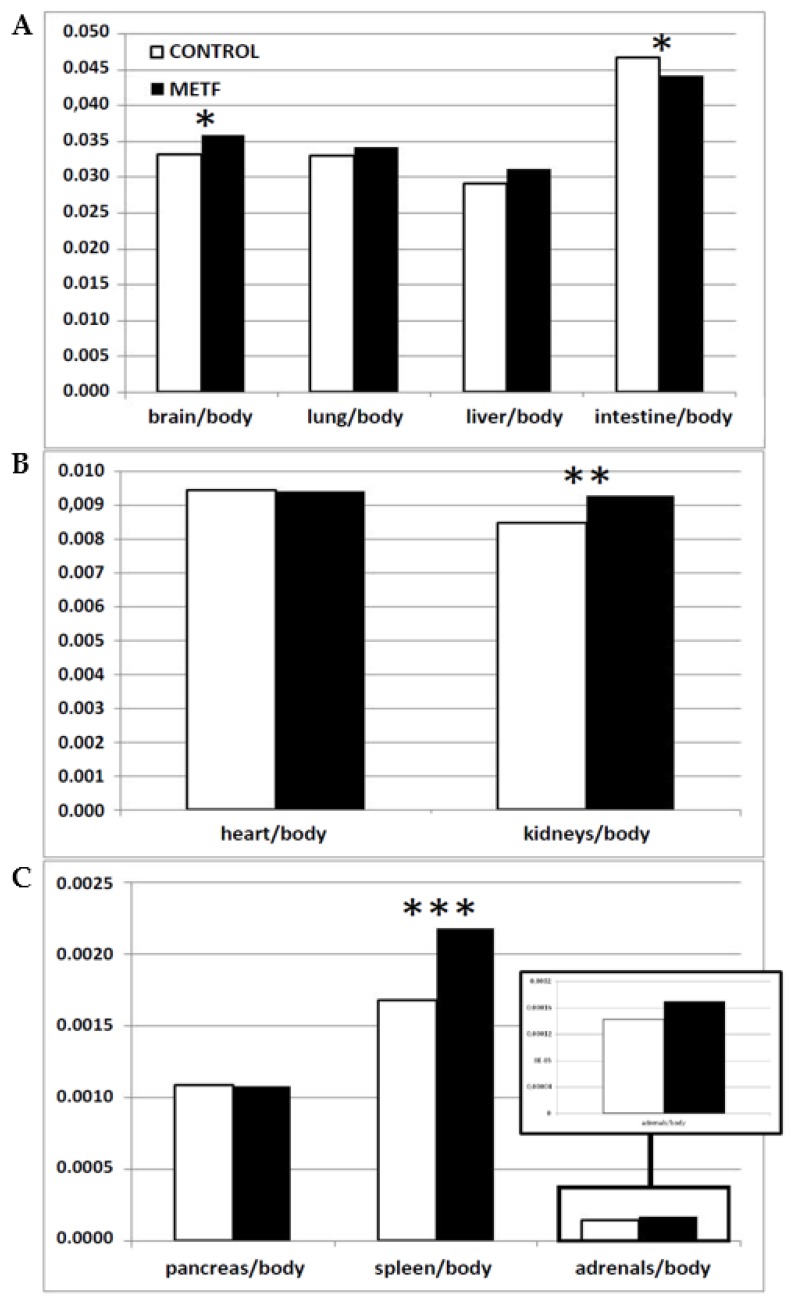
Mean values of weight ratios of brain, lung, liver, and intestine to total-body-weight (**A**), heart and kidneys to total-body-weight (**B**) and pancreas, spleen, and adrenals to total-body-weight (**C**) in controls (white bars) and maternal-metformin-treated fetuses (black bars). Asterisks denote significant differences (* = *p* < 0.05, ** = *p* < 0.005, *** = *p* < 0.0005).

Analysis of weight ratios among different organs and total body weight (Figure 1) showed that METF fetuses had significantly higher values for brain/body-weight (*p* < 0.05), kidneys/body-weight (*p* < 0.005), and spleen/body-weight (*p* < 0.0005), and trends for higher liver and adrenals/body-weight (*p* = 0.08 for both). Conversely, the ratio intestine/body-weight was higher in controls (*p* < 0.05).

The maternal metformin therapy induced significant increases in the fetal plasma concentration of parameters indicative of glycemic (glucose and fructosamine, *p* < 0.005 and *p* < 0.0005, respectively) and lipid profile (triglycerides, *p* < 0.0005), without significant changes in total-, HDL-, and LDL-cholesterol), as shown in the Table 3.

## 3. Discussion

The present study is the first trial (to the best of our knowledge) supporting that maternal treatment with metformin during pregnancy improves fetal development and metabolism in pregnancies not affected by obesity and/or diabetes but by undernutrition. These outcomes were independent of any effect on maternal traits. In fact, there were no differences in body-weight, fatness, and metabolic features between control and treated sows; such findings are in agreement with previous data evidencing no weight-gain or overt hypoglycemia in human beings exposed to long-term metformin treatments [5,12].

In spite of any significant effect on maternal features, the treatment improved fetal traits. Metformin increased the weights of fetal entire body and carcass (trunk without viscera), but these differences were not found to be statistically significant. Such a lack of differences between groups may be related to the stage of pregnancy in which fetuses were sampled (day 100; around 90% of swine pregnancy), since fetal development follows an exponential curve with a quadratic increase from day 45 to 115 of pregnancy, in which the greatest increase occurs during the last 10% of the pregnancy (15 last days of gestation) [13]. Hence, further studies on at-term pregnancies or birth are needed. On the other hand, a significantly positive effect of metformin treatment was found when considering the fetal viscera. Fetuses in the treated group showed a higher weight of the brain and the total thoracic and abdominal viscera (mainly due to higher weights of liver, kidneys, spleen, and adrenal glands). These findings suggest a protective effect of the treatment on the developmental competence of the fetuses. 

The occurrence of a challenge compromising fetal growth and developmental competence causes asymmetrical IUGR. Asymmetrical IUGR, depending on the pregnancy stage and the severity of the challenge, is characterized by the prioritization of the growth of brain, liver, and adrenal glands (or only the brain in the most severe processes) at the expense of the growth of body (bones and skeletal muscles) and other organs. The growth of the brain is protected by a redistribution of the fetal blood flow (the “brain-sparing effect” firstly described by Rudolph in 1984 [14]) for assuring the survival of the offspring after birth. A failure in the supply of oxygen and/or nutrients to the developing brain may cause adverse neurological outcomes [15,16], which may compromise critical functions such as breathing, suckling, and/or any of the so-called autonomic functions [17]. In the current study, the growth of brain and head was prioritized, but an excessive growth related to macrocephaly can be discarded, since values were within a normal range for the breed and the gestational age. The other organ in which growth was prioritized was the liver, due to “liver-sparing” cardiovascular adaptations similar to the “brain-sparing effect” [18,19]. The liver is also essential for offspring viability. Hence, neonates are strongly dependent on glycogen deposition and gluconeogenic capacity of the organ to allow the transition from a continuous supply of nutrients from the placenta to the intermittent supply coming from suckled milk [20,21]. Finally, it is also well-known that growth of adrenal glands is increased in restricted fetuses [22]. In the current work, in a similar way to brain and liver, the metformin treatment favored the growth of adrenal glands. In summary, our results indicate that maternal metformin treatment may have a positive effect protecting the adequate growth and development of the brain, liver, and adrenal glands, and we therefore suggest that the metformin treatment may favor vitality and survival of the neonate.

The growth of brain and liver is protected, in the case of IUGR, at the expense of other organs—mainly lungs, kidneys, and spleen [18]—which imposes important consequences on the adequate function of these organs at postnatal stages. Adequate lung development is essential during the very early postnatal stages, but fetal growth retardation results in permanent changes in lung structure [23], and offspring that are small for gestational age commonly have compromised lung function and increased respiratory morbidity into adulthood [24]. In the current study, the supply of metformin to pregnant sows induced a trend for a better development of the lungs, although differences did not reach statistical significance. The renal system is also affected by fetal growth retardation, which may impair nephrogenesis, causing a decrease in the glomerular number and a compensatory glomerular enlargement. The consequences are reduced nephron endowment, hypertension, and renal diseases in adulthood [25,26,27]. The results of the present study indicate that the metformin treatment prevents the decrease in kidney size, characteristic of IUGR fetuses and indicative of changes in renal morphology, and set the basis for further studies assessing the effects of metformin on renal development and function—a main target, since renal function is strongly affected in offspring from risk pregnancies, causing chronic kidney disease and cardiovascular (hypertension) diseases in adulthood [26,28]. The same applies for the spleen, since there is evidence of a deleterious effect of IUGR processes on spleen development, which would contribute to susceptibility of IUGR neonates to infections [29].

In summary, the maternal treatment with metformin favored the development of most of the main fetal organs except the intestine. The adequate development of the intestine is also essential for the offspring during the first stages of life, since it favors absorption and utilization of nutrients and other substances such as immunoglobulins [30]. However, in the conditions of the present study, we cannot elucidate why its development was not favored in a similar way to the other organs in treated offspring. 

Our results suggest that the positive effects of maternal metformin treatment on fetal developmental competence may be, at least in part, mediated by an improved placental development apart from direct effects on the fetus, since metformin crosses the placenta [4]. In our study, the assessment of placental weight showed a trend for higher values in the treated group. We can hypothesize for further studies that the better placental development in treated animals may be based in the up-regulatory effect of metformin on receptors for vascular endothelial growth factor (VEGFR1 and VEGFR2 [31]). The VEGF ligand-receptor system is a specific stimulator of adaptive changes for the establishment of angiogenesis, the maintenance of adequate vascularization at implantation sites, and the later development of placenta [32,33].

A better placental growth and function would increase the supply of nutrients and oxygen to the fetus, which may counteract IUGR occurrence and consequences. Our hypothesis may be reinforced by the higher availability of glucose, fructosamine (indicative of precedent glucose availability), and triglycerides in the fetuses from the treated group. Glucose, which crosses the placenta via facilitated transport, and triglycerides, which are previously hydrolyzed by placental lipases to fatty acids, are the main sources of energy for developing fetuses [34,35,36]. The increase of glucose in treated fetuses supports the initial hypothesis of the current study about a better uptake and use of glucose by fetuses exposed to metformin. Increased fetal triglycerides may indicate a better transfer of fatty acids by the placenta and a better fetal metabolism, since fatty acids are used by fetuses for triglyceride synthesis.

In summary, the results obtained in the current study support a beneficial effect of the metformin treatment on fetal development and metabolism. However, as recently stated, there are no long-term safety data for the use of the drug during pregnancy [37], thus it should not be used as a first-line agent prior to a profound study of the long-term outcomes in the offspring. Moreover, several studies have shown evidence of a higher size, weight, and body mass index (BMI) of metformin-exposed children [38,39] as well as the negative consequences on sociability [40] and reproductive function [41] in metformin-exposed rodents.

## 4. Material and Methods

### 4.1. Animals and Ethics Statement

The study involved 47 near-to-term fetuses, which were obtained from six purebred Iberian sows. The sows became pregnant after cycle synchronization with altrenogest (Regumate^®^, MSD, Boxmeer, The Netherlands) and insemination with cooled semen from a purebred Iberian boar. These females were housed at the INIA animal facilities, which meet local, national, and European requirements for Scientific Procedure Establishments. The study was performed according to the Spanish Policy for Animal Protection RD53/2013, which complies with the European Union Directive 2010/63/UE on the care of animals used for research, and the experimental procedures were assessed and approved by the INIA Committee of Ethics in Animal Research and the local authorities (report PROEX 353/15).

### 4.2. Animal Handling and Experimental Procedure

The sows were fed with a standard grain-based food diet with the following mean component values: dry matter, 89.8%; crude protein, 15.1%; fat, 2.8%; and metabolizable energy, 3.0 Mcal/kg. The amount of food was adjusted to fulfill individual daily maintenance requirements from the start of the experimental period to day 35 of pregnancy. This diet was reduced to fulfill only 50% of the individual daily requirements from day 35 of pregnancy to day of fetal sampling (day 100 of pregnancy) in order to impose a nutritional challenge and to induce a higher incidence of IUGR processes [10,11]. The day 100 of pregnancy, corresponding to approximately 90% of a 112-days gestation length typical for this breed, was chosen for sampling because fetal metabolism becomes independent from maternal signals to be directly affected by nutrient availability from day 90 onwards [42].

On the same day 35 of pregnancy, the sows were pair-matched according to body-weight, with half of them receiving no treatment and therefore acting as the untreated control group (group C), whilst the remaining females (group METF) were treated by receiving 850 mg of metformin per animal and day (Dianben^®^; Merck Serono, Madrid, Spain) by individually top-dressing over the morning feed from day 35 to day of sampling. The dose of metformin was chosen based on previous studies with the same breed [7]. At day 35 of pregnancy, there were no differences between females allocated to the groups C and METF in body weight (101.5 ± 4.7 and 104.7 ± 5.6 kg, respectively) or back-fat depth, which was determined by ultrasonography (5–8 MHz lineal-array probe, SonoSite Inc., Bothell, WA, USA) at the right-side at 4 cm from the midline and the head of the last rib (22.5 ± 3.8 and 23.2 ± 2.4 mm, respectively).

At day 100 of pregnancy, after fasting for approximately 16 h, body-weight and back-fat depth were evaluated in all the sows again, and blood samples were drawn from the orbital sinus. These samples were collected in sterile, heparinized 4 mL vacuum tubes (Vacutainer™ Systems Europe, Meylan, France) and were immediately centrifuged at 1500 × g for 15 min. The plasma was separated and biobanked into polypropylene vials at −80 °C until they were assayed for indices of glucose and lipids metabolism.

### 4.3. Measuring, Weighting and Sampling of Fetuses

The sows were sequentially euthanized by stunning and exsanguination at day 100, in compliance with RD53/2013 standard procedures. In each sow, the entire genital tract was immediately collected for morphometric evaluation and sampling of fetuses; sex was determined by visual inspection immediately after recovery. At once, for each fetus, blood samples were drawn from the heart using heparinized syringes and processed as described above for sows. Body-weight and -length (crown-rump length), head size (occipito-nasal length and biparietal diameter), and corpulence (thoracic and abdominal circumferences) were measured in all the fetuses. Then, the head was separated from the trunk at the atlanto-occipital union and weighed separately for determining the ratio of head-to-body weight. All viscera were obtained and weighed together immediately, and then brain, heart, lungs, liver, intestine, kidneys, spleen, pancreas, and adrenal glands were weighed separately for assessing patterns of asymmetrical IUGR. Finally, placentas were individually isolated and weighed.

### 4.4. Evaluation of Maternal and Fetal Metabolic Status

Parameters for the glycemic index (glucose, fructosamine) and the lipid profile (triglycerides, total cholesterol, HDL-c, and LDL-c) were measured in both maternal and fetal plasma. Assays were performed using a clinical chemistry analyzer (Saturno 300 plus, Crony Instruments s.r.l., Rome, Italy) according to the manufacturer’s instructions.

### 4.5. Statistical Analyses

Data were analyzed using SPSS 22.0 (IBM, New York, NY, USA). T-student tests were used to assess the effects of maternal treatment (control versus treated) on litter size and distribution of sexes. Effects of treatment (control versus treated) and sex (female versus male) on developmental and metabolic traits were assessed by two-way ANOVA. Duncan’s post-hoc test was performed to check differences among groups in multiple comparisons. All results were expressed as mean ± SEM, and statistical significance was accepted from *p* < 0.05, while *p*-values between 0.05 and 0.09 were considered to indicate a tendency.

## 5. Conclusions

Our results, obtained in a swine model of IUGR pregnancies, support the use of metformin for enhancing metabolic traits and developmental competence of fetuses from undernourished mothers with risk pregnancies. Obviously, metformin treatment should not be a replacement for nutrition in the patients who do not have a medical condition. These findings may be of high value for human medicine not only in cases of maternal malnutrition, since metformin is a cheap drug easily available, but also in cases of placental deficiency, since metformin seems to improve placental development and function. However, further studies are necessary to determine the effects of maternal metformin treatment on the postnatal development, the health/disease status of the offspring, and its real translational value for human beings.

## Figures and Tables

**Table 1:** Mean (± SEM) values for body-weight (kg), back-fat depth (mm), and plasma parameters (mg/dL) of glucose, fructosamine, and lipids [triglycerides and total-, high-density lipoproteins cholesterol (HDL-c), and low-density lipoproteins cholesterol (LDL-c)] in control (group C) and metformin-treated sows (group METF) at day 100 of pregnancy.

		Group C	Group METF
Body morphometry	Body weight	111.2 ± 1.1	113.8 ± 2.1
	Back-fat depth	21.7 ± 5.0	24.4 ± 5.0
Glucose metabolisms	Glucose	90.3 ± 0.8	85.3 ± 1.2
	Fructosamine	302.1 ± 4.0	266.7 ± 2.6
Lipid metabolisms	Triglycerides	52.3 ± 2.4	57.7 ± 2.1
	Total cholesterol	58.6 ± 1.7	54.9 ± 1.0
	HDL-cholesterol	25.6 ± 1.0	23.2 ± 0.7
	LDL-cholesterol	31.2 ± 1.5	33.1 ± 1.6

**Table 2:** Mean (± SEM) values for fetal size, total-body-weight, and weight of different organs and structures in control (group C) and metformin-treated fetuses (group METF) at day 100 of pregnancy. The third column represents the percentage of increase in the values when comparing group METF to group C.

	Group C	Group METF	Increase (%)
Body weight (g)	756.9 ± 28.1	806.7 ± 25.8	6.6
Body length (cm)	21.4 ± 0.3	21.1 ± 0.4	0.0
Occipito-nasal length (cm)	11.1 ± 0.2	11.1 ± 0.3	0.0
Biparietal diameter (cm)	4.4 ± 0.1	4.5 ± 0.1	2.3
Thoracic circumference (cm)	18.3 ± 0.3	17.8 ± 0.3	0.0
Abdominal circumference (cm)	14.0 ± 0.3	13.9 ± 0.3	0.0
Head weight (g)	171.6 ± 5.1	178.1 ± 5.3	3.8
Carcass weight (g)	428.6 ± 17.4	445.3 ± 15.9	3.9
Brain weight (g)	25.5 ± 0.5 ^g^	28.1 ± 0.4 ^h^	10.2
Total viscera weight (g)	122.0 ± 6.7 ^c^	137.5 ± 4.7 ^d^	12.7
Heart weight (g)	7.2 ± 0.4	7.4 ± 0.3	2.8
Lungs weight (g)	24.9 ± 1.1 ^a^	27.7 ± 1.1 ^b^	11.2
Liver weight (g)	22.2 ± 1.0 ^c^	25.4 ± 1.2 ^d^	14.4
Intestine weight (g)	35.4 ± 1.5	35.1 ± 1.3	0.0
Kidneys weight (g)	6.4 ± 0.3 ^c^	7.4 ± 0.3 ^d^	15.6
Spleen weight (mg)	129.1 ± 7.7 ^g^	172.1 ± 7.4 ^h^	33.3
Pancreas weight (mg)	834.9 ± 5.3	862.6 ± 5.5	3.3
Adrenal glands (mg)	105.5 ± 5.5 ^c^	134.4 ± 1.0 ^d^	27.4
Placental weight (g)	253.2 ± 12.7 ^a^	286.2 ± 12.6 ^b^	13.0

Superscript letters denote significant differences: (a ≠ b: 0.09 > *p* > 0.05; c ≠ d: *p* < 0.05; e ≠ f: *p* < 0.005; g ≠ h: *p* < 0.0005).

**Table 3:** Mean (± SEM) values for plasma concentrations (mg/dL) of glucose, fructosamine, and lipids (triglycerides and total-, HDL-, and LDL-cholesterol) in control (group C) and metformin-treated fetuses (group METF) at day 100 of pregnancy.

	Group C	Group METF
Glucose	176.1 ± 3.6 ^e^	260.3 ± 2.4 ^f^
Fructosamine	126.1 ± 5.1 ^g^	189.8 ± 7.1 ^h^
Triglycerides	48.6 ± 3.8 ^g^	67.5 ± 4.3 ^h^
Total cholesterol	53.7 ± 3.1	56.9 ± 2.3
HDL-cholesterol	25.2 ± 2.7	17.5 ± 0.7
LDL-cholesterol	33.5 ± 2.1	34.2 ± 1.6

Superscript letters denote significant differences: (e ≠ f: *p* < 0.005; g ≠ h: *p* < 0.0005).
